# C-reactive Protein Kinetics During In-Patient Treatment of COVID-19-Associated Rhino-Orbito-Cerebral Mucormycosis: A Retrospective Cohort Study in a Tertiary Hospital in Central India

**DOI:** 10.7759/cureus.59007

**Published:** 2024-04-25

**Authors:** Shrikrishna B. H., Vijay Bidkar, Kirankumar Prathipati, Sandeep Dabhekar, Kalaiselvi Selvaraj, Deepa G.

**Affiliations:** 1 Otolaryngology - Head and Neck Surgery, All India Institute of Medical Sciences, Bibinagar, Hyderabad, IND; 2 Otolaryngology - Head and Neck Surgery, All India Institute of Medical Sciences, Nagpur, Nagpur, IND; 3 Community Medicine, All India Institute of Medical Sciences, Madurai, Madurai, IND; 4 Anatomy, All India Institute of Medical Sciences, Bibinagar, Hyderabad, IND

**Keywords:** virus, sinusitis, serum, pandemic, mucormycosis, c-reactive protein, covid-19

## Abstract

COVID-associated rhino-orbito-cerebral Mucormycosis (CA-ROCM), henceforth referred to as Covid-Associated Mucormycosis (CAM), is a serious and fatal condition unless treated promptly and completely. The main treatment of the CAM is complete surgical debridement and administration of systemic antifungals. The first line antifungal recommended for CAM is Amphotericin-B. Since Amphotericin-B has systemic side effects mainly on the renal system, a timely decision to start and end Amphotericin-B therapy is very essential. Besides the Computed Tomography (CT) scan, serum levels of C-reactive protein (CRP) levels are a good indicator of CAM-associated inflammation levels in the patient's body. By monitoring the CRP levels, we can titrate amphotericin treatment to cause minimal harm to the kidneys. Our study was done to analyze the kinetics of C-reactive protein in patients of CAM admitted in a tertiary-care hospital and compare it with the CRP levels in COVID-associated non-Mucormycosis Sinusitis patients.

Aim and objective

To study the kinetics of serum C-reactive protein (CRP) levels among patients undergoing in-patient care for COVID-associated rhino-orbito-cerebral mucormycosis and compare with serum CRP levels in COVID-19 patients suffering from sinusitis without rhino-orbito-cerebral mucormycosis.

Materials and methods

This was a retrospective cohort study. The source of data was post-COVID sinusitis patients who were admitted during 2nd wave of COVID-19 in India in our hospital whose medical records were accessed by the Medical Records Department. The subjects were recruited into the two study groups namely the Mucormycosis group and the non-Mucormycosis group based on the histopathological report of the nasal biopsy specimen. The medical records of each member of the two groups were studied for the levels of serum C-reactive protein measured at the time of admission and every 5(+1) days thereafter till the time of discharge. The kinetics of serum C-reactive protein levels, which is a marker of inflammation is studied in each of the two groups and compared using statistical methods.

Results

There was a significant difference between Mucormycosis and Non-Mucormycosis groups in CRP-level kinetics. However, there was no significant trend of decrease or increase over time in Mucormycosis as well as non-Mucormycosis cases.

Conclusion

CRP is an important biomarker in assessing the septic response to COVID-associated rhino-orbito-cerebral mucormycosis. Detection of raised CRP levels helps in prompt early initiation of anti-fungal treatment. Also, monitoring the levels of serum CRP will guide in deciding the time to stop the antifungals at an appropriate time. CRP monitoring is commonly available and affordable. Hence, we recommend CRP monitoring of in-patients of CAM.

## Introduction

The novel severe acute respiratory syndrome coronavirus-2 (SARS-CoV-2) has been a global pandemic. The virus causes inflammation of the respiratory tract, and eventually ground-glass opacity of the lungs. Due to severe inflammatory reactions and diffuse alveolar damage, COVID-19 patients have a decline in their CD-4+ and CD-8+ T cell count, making them liable to a wide variety of infections, notably fungal infections. Mucormycosis (also known as zygomycosis) is a dangerous fungal infection caused by mucoromycetes, a type of mold. There have been more mucormycosis instances among COVID-19 patients worldwide, notably in India. This is because the environment in a sick person is favorable for the growth of spores, including hypoxia, elevated glucose levels brought on by diabetes or steroid-induced hyperglycemia, increased ferritin levels, an acidic environment brought on by metabolic acidosis or diabetic ketoacidosis, and a decline in the activity and quantity of white blood cells [1,2]. 

Amphotericin B is the first-line therapy for COVID-associated mucormycosis (CAM). Amphotericin B toxicity causes metabolic imbalances and renal problems. Triazoles, such as posaconazole and isavuconazole, which inhibit ergosterol formation in the fungal cell membrane, should be begun in those with impaired renal function. However, Posaconazole-induced severe hyperbilirubinemia is an uncommon form of drug-induced liver injury that has yet to be documented in case reports. Hence, to ensure the least harm due to the side effects of antifungals during the in-patient care of Covid-Associated Mucormycosis, disease-associated inflammation has to be monitored regularly. Very elevated C-reactive protein (CRP) values in immunocompromised individuals may signify a systemic fungus infection. The use of this knowledge could simplify the diagnosis procedure [3,4]. Deep-seated fungi diseases like Mucormycosis are linked to high levels of CRP in terms of their capacity to cause high (> 100 mg/L) values [5]. Amphotericin-B is started empirically in a suspected case of CAM as a life-saving measure, but the stopping of its administration can be decided once the CRP levels show a decreasing trend or become normal. This will save the patient from the cumulative renal side effects of Amphotericin-B. By monitoring the CRP levels, we can titrate Amphotericin treatment to cause minimal harm to the kidneys. There are limited studies on the kinetics of CRP or any other inflammatory marker during the in-patient treatment of CAM. Hence, our study aims to study the kinetics of serum CRP levels in patients undergoing in-patient care for CAM and compare them with serum CRP levels in COVID patients suffering from sinusitis without mucormycosis. 

Objective

To study the kinetics of serum CRP levels in patients undergoing in-patient care for CAM and compare them with serum CRP levels in post-COVID patients suffering from sinusitis without mucormycosis. 

## Materials and methods

This study was a retrospective cohort conducted at All India Institute of Medical Sciences, Nagpur. The study was initiated after the due approval of the Institute Ethics Committee. The study was done according to Good Clinical practice guidelines. The study was conducted between June 2022 to September 2022. The case records of COVID patients admitted to the ENT department for the management of sinusitis from May 2021 to October 2021 during 2nd wave of COVID-19 in India were obtained from the Medical Records department. The subjects were recruited into the two study groups namely the Mucormycosis group and the non-Mucormycosis group based on the following inclusion and exclusion criteria: 

Inclusion Criteria for COVID-associated mucormycosis group: 1. COVID-positive RT-PCR report within the past 6 months from the onset of symptoms of sinusitis. 2. Sinusitis was confirmed by computed tomography of the paranasal sinuses. 3. Mucormycosis was confirmed by a histopathology report of nasal tissue showing broad aseptate fungal hyphae with tissue damage.

Exclusion Criteria for COVID-associated mucormycosis group: 1. Mucormycosis was ruled out by a histopathology report of nasal tissue.

Inclusion Criteria for COVID-associated non-mucormycosis group: 1. COVID-positive RT-PCR report within the past 6 months from the onset of symptoms of sinusitis. 2. Sinusitis was confirmed by computed tomography of the paranasal sinuses. 3. Mucormycosis was ruled out by a histopathology report of nasal tissue.

Exclusion Criteria for COVID-associated non-mucormycosis group: 1. Mucormycosis was confirmed by a histopathology report of nasal tissue showing broad aseptate fungal hyphae with tissue damage.

Both the groups had undergone surgical debridement of the paranasal sinuses within 2 days of admission after pre-operative anesthesia fitness workup. The group with histopathologically confirmed Mucormycosis was started on systemic amphotericin-B postoperatively administered for an average duration of 14 days. The group with no histopathological report of mucormycosis did not receive systemic amphotericin-B. The medical records of each member of the two groups were studied for the levels of serum C-reactive protein measured at the time of admission and every 5(+1) days thereafter till the time of discharge. The kinetics of serum C-reactive protein levels are studied in each of the two groups and compared using statistical methods. The collected data were analyzed statistically. A p-value of 0.05 or below was considered statistically significant.

## Results

During our study period, a total of 35 patients presented to us with sinusitis, and all of them had a history of COVID-19 positivity in the previous 6 months. Among them, nineteen patients had histopathological confirmation of mucormycosis and were included in the Mucormycosis group. Sixteen patients were negative for mucormycosis according to histopathology and were included in the non-Mucormycosis group. The mean age of the patients in the Mucormycosis group was 55.8 years (SD 14.3), while it was 50.1% (SD 11.7) in the non-Mucormycosis group. 11 men and eight women made up the Mucormycosis group. Eight men and eight women made up the non-Mucormycosis group. There were 12 known diabetics receiving therapy in the Mucormycosis group. Only 6 known diabetics receiving therapy belonged to the non-Mucormycosis group. At presentation, the Mucormycosis group's random blood sugar level was 183.2 mg%. The random blood sugar level at presentation was 143.9 mg% in the non-Mucormycosis group. HbA1C level at presentation was 7.2 in the Mucormycosis group. The HbA1C level at presentation was 6.7 in the non-Mucormycosis group.

The patients' case records revealed the values of serum CRP levels were studied in each of the two groups. It was noted that serum CRP levels were assessed on different days of the in-patient stay, for example, on day 16, CRP measurements were available for only four patients. Hence, values on all days could not be considered. Hence, we considered day 1, day 5, and day 15 values which gave the sample size of 28 in total. In the final 28 total sample size, 17 patients belonged to the Mucormycosis group and 11 patients belonged to the non-Mucormycosis group.

Table 1 shows the mean CRP levels of patients in each group on days 1, 5, and 15. The mean CRP level on Day 1 was 36.4 (SD=29.8) in the Mucormycosis group and 6.4 (SD=5.3) in the non-Mucormycosis group. The mean CRP level on Day 5 was 51.8 (SD=37) in the Mucormycosis group and 20 (SD=23.3) in the non-Mucormycosis group. The mean CRP level on Day 15 was 52.3 (SD=36.5) in the Mucormycosis group and 12 (SD=12.1) in the non-Mucormycosis group. 

**Table 1:** Distribution of CRP values among patients admitted for mucormycosis and non-mucormycosis sinusitis CRP: C-reactive protein

Day / CRP Levels (mg/L)	Mucormycosis	(n=)	Non-Mucormycosis Sinusitis	(n=)
Day 1	36.4 (29.8)	17	6.4 (5.3)	11
Day 5	51.8 (37)	17	20 (23.3)	11
Day 15	52.3 (36.5)	17	12 (12.1)	11

Table 2 shows the median and Interquartile range of CRP levels of patients in each group on days 1, 5, and 15. The median CRP level on Day 1 was 14.4 in the Mucormycosis group with an Interquartile range of 33.1-61.3. The median CRP level on Day 1 was 5.2 in the non-Mucormycosis group with an Interquartile range of 3.1-7.4 (p =0.001). The median CRP level on Day 5 was 19.1 in the Mucormycosis group with an Interquartile range of 47.6-81. The median CRP level on Day 5 was 10.3 in the non-Mucormycosis group with an Interquartile range of 3.3-20.5 (p =0.005). The median CRP level on Day 15 was 21.8 in the Mucormycosis group with an Interquartile range of 38.6-82.5. The median CRP level on Day 15 was 9.4 in the non-Mucormycosis group with an interquartile range of 7.4-11.5 (p =0.002). The median CRP values among patients with Mucormycosis was significantly higher compared to patients with non-Mucormycosis across various follow-up period time. However, there was no significant trend observed within the Mucormycosis group or non-Mucormycosis group of patients over different follow-up periods. 

**Table 2:** Non-parametric comparison of CRP values among patients admitted for mucormycosis and non-mucormycosis sinusitis CRP: C-reactive protein

	Day 1	Day 5	Day 15	Significance
CRP values (mg/L)	Median (IQR)	Median (IQR)	Median (IQR)	(Trend in CRP values during follow-up)
Mucormycosis (n=17)	14.4 (33.1-61.3)	19.1 (47.6-81.)	21.8 (38.6-82.5)	0.42
Non-Mucormycosis (n=11)	5.2 (3.1-7.4)	10.3 (3.3-20.5)	9.4 (7.4-11.5)	0.09
Comparison of CRP values Mucormycosis Vs Non-Mucormycosis	0.001	0.005	0.002	

**Figure 1:**
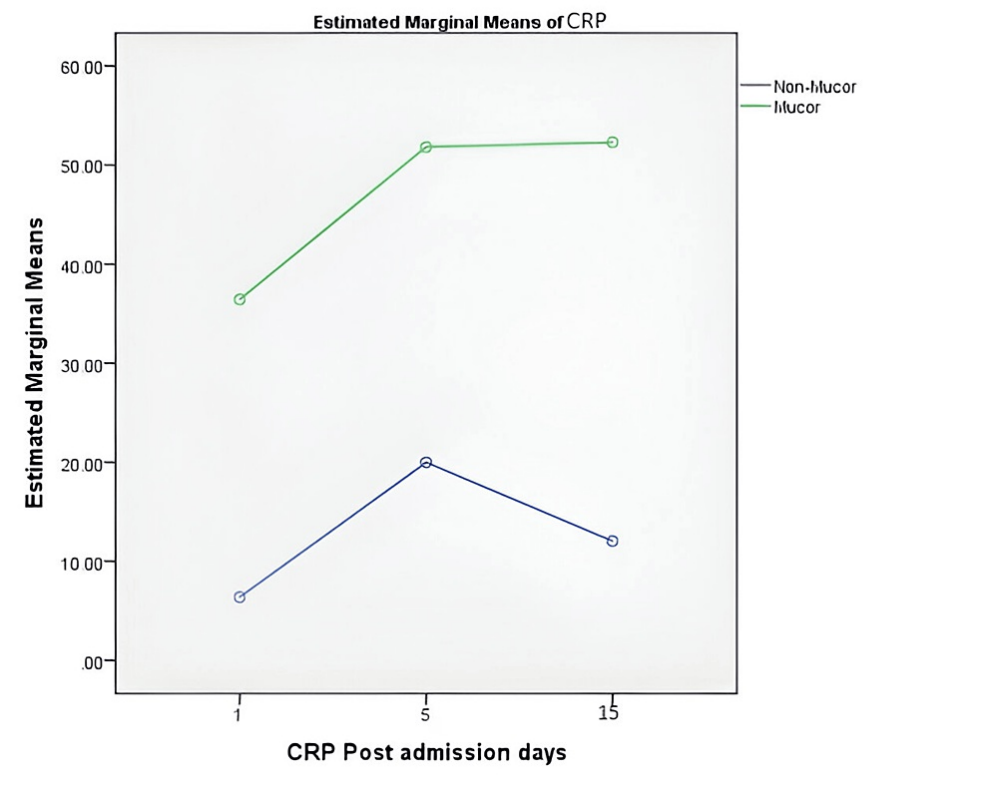
Comparison of marginal means of CRP values across days 1, 5, and 15 among mucormycosis vs non-mucormycosis cases CRP: C-reactive protein

Figure 1 shows the marginal means of CRP in each group on days 1, 5, and 15. As is evident from the figure, The marginal means of CRP values among patients with Mucormycosis was higher compared to patients with non-Mucormycosis across various follow-up periods.

## Discussion

In March 2020, the new severe acute respiratory syndrome coronavirus-2 (SARS-CoV-2) was designated a pandemic [1,2]. Coughing, fever, and dyspnea are symptoms of the sickness, along with a change in taste and smell, urticaria and skin rashes, and, in severe instances, altered consciousness, disorientation, and stroke [6,7]. It finally results in ground-glass opacity in the lungs [8]. Patients who have low CD-4+ and CD-8+ T cell counts are more vulnerable to fungus infections [9]. Mucormycosis is a deadly fungal illness caused by *Rhizopus*, *Rhizomucor*, *Mucor*, *Syncephalastrum*, *Apophysomyces*, *Absidia*, and *Cunninghamella*. *Rhizopus oryzae *is the most common type, accounting for 90% of rhino-orbital-cerebral mucormycosis cases (ROCM) [10,11,12]. During the second wave of the epidemic, India emerged as a hotspot for COVID-mucormycosis, with over 20000 cases recorded [13]. Hypoxia, raised glucose levels owing to diabetes or steroid-induced hyperglycemia, increased ferritin levels, and a reduction in the activity and quantity of white blood cells were the culprits [14].

Amphotericin B, the first-line therapy for mucormycosis, results in metabolic irregularities and renal problems. Triazoles, such as posaconazole and isavuconazole, the second-line treatment, which can induce hyperbilirubinemia, should be begun in those with impaired renal function. As a result, disease-associated inflammation must be evaluated frequently to ensure that no harm is caused by antifungal side effects during in-patient therapy for mucormycosis [3, 4]. Even though CRP is a non-specific inflammatory marker, deep-seated fungal illness, such as mucormycosis, is associated with elevated levels of CRP 100-300 mg/L, especially in immunocompromised people [5]. Similar reports were shared also by Montagna et al who state that elevated CRP levels may suggest an invasive fungal infection and, as a result, prompt the commencement of specific medication in both non-neutropenic and neutropenic patients. [15].

Because there is no definitive technique of diagnosis, mucormycosis diagnosis is sometimes delayed or difficult to establish with certainty. Clinical signs vary, are non-specific, and may emerge only after the infection has spread. Tissue biopsies are the top standard, although they are not always feasible. Roques M et al have proposed in their study that mucormycosis is accompanied by a significant rise in serum CRP and fibrinogen levels [16]. Besides cytopenia, several non-specific indicators linked with COVID-19-related sepsis, such as CRP, D-dimer levels, and lactate dehydrogenase are reported to be disturbed in cases of combined mucor infection, in addition to a dysfunctional renal profile [17].

CRP is a small component of serum with a median value of 0.58 mg/L in healthy people [18]. It increases to 300 mg/l within 24-48 hours as a non-specific response, depending on the degree of the injury, whether surgical or inflammatory. Hepatocytes are likely to be activated during surgery, and they can create CRP in just six hours and alter levels for several days [19]. This post-surgical rise in levels of CRP explains the rise in levels of CRP in our patients of the Mucormycosis group who underwent surgical debridement of the nose and paranasal sinuses for mucormycosis. CRP is an important biomarker in assessing the septic response to COVID-associated rhino-orbito-cerebral mucormycosis. Kostiala has stated that CRP determination may contribute to the early detection of fungal sepsis [20]. Early detection should be promptly followed with early initiation of anti-fungal treatment. Healthcare workers should also keep a watch on linked co-morbidities to detect Mucormycosis at the earliest. According to Sercan Gode et al. and Cho HJ et al., high CRP level is associated with poor prognosis in mucormycosis [21, 22]. Although Cho HJ et al. discovered that a threshold CRP level of 5.50 mg/dL best predicted outcomes, Sercan Gode et al. discovered that a cutoff CRP level of 4 mg/dL had 94.1% sensitivity and 47.1% specificity. Shastri et al. have opined in their study that a high CRP level (above 100) is associated with poor outcomes in CAM [23]. In their study, a significant 65.38% of patients had a very high level of CRP. However, we could not establish the same in our study as all of our patients in the Mucormycosis group recovered completely.

As in the study by Shubashree Karat et al., CRP levels were considerably higher in the Mucormycosis group compared to the control group, and greater levels of CRP have been related to an increased risk of developing COVID-19-associated Mucormycosis. [24]. In our study also, a similar finding was observed on day 1 of admission the mean serum CRP level was 36.4 mg/L in the Mucormycosis group and 6.4 mg/L in the non-Mucormycosis group. Thus, a high index of suspicion with a raised CRP level should be used to initiate empirical anti-fungal treatment till a proper histopathological report is procured. During the early stages of COVID-19 illness, CRP, an acute phase protein generated from hepatocytes as a result of interleukin (IL) 6 dysregulation, is a potent predictor of disease progression and poor outcomes [25]. With the usage of receptors such as glucose-regulated protein 78 (GRP78), higher levels of inflammation can reduce pH, encourage hyperglycemia (lowering normal immunological activity), and impede endothelial function [26, 27]. The serum CRP levels in all of our Mucormycosis cases were high with a mean value of 36.4 mg/L. Whether high CRP levels contributed to the development of mucormycosis or mucormycosis led to high serum CRP is a matter of research.

There is a general rise in serum CRP levels post-operatively after any major surgery. In the study by Santonocito et al., over the first few days following surgery, CRP levels rose in all patients, but throughout the first seven days, they were greater in infected patients than in noninfected patients [28]. They recommend that repeated CRP measurements may be used to determine the length of antibiotic therapy, or surgical re-exploration may be considered if predicted declines in CRP levels are not observed. All the mucormycosis patients in our study underwent surgical debridement of the nose and sinuses between the 1st and 5th day, based on their fitness for surgery. The patients of the non-Mucormycosis group underwent functional endoscopic sinus surgery between the 1st and 5th day, based on their fitness for surgery. The serum CRP levels decreased in non-Mucormycosis cases after the fifth postoperative day. In the Mucormycosis group, the CRP levels flattened or decreased slowly with time. This difference in the kinetics of serum CRP levels hints at the role of mucormycosis infection in increasing CRP levels. 

In the study by Shenoy et al., the postoperative recovery of patients was evaluated using C-reactive protein levels. Treatment failure was assumed when CRP levels were increased continuously for 4 days [29]. In our study also, postoperative patient recovery was evaluated using C reactive protein levels. All the mucormycosis patients in our study underwent surgical debridement of the nose and sinuses between the 1st and 5th day, based on their fitness for surgery. All of our patients began receiving intravenous liposomal amphotericin B (5-10 mg/kg) for 2-4 weeks, and once the blood CRP level had stabilized, they continued receiving oral posaconazole (600 mg on the first day and 300 mg daily from the second day) for 3 months. Between the 5th and 15th day of the in-patient stay, the CRP level flattened or decreased slowly with time and all the patients in the Mucormycosis group recovered completely without any fatality. Hence, we recommend monitoring serum CRP among in-patients of mucormycosis on a global standard after further studies on a larger scale.

The small patient population and single facility setting are two of the study's drawbacks. Also, our research was undertaken during a mucormycosis outbreak and the worldwide COVID-19 pandemic. As a result, our findings are not typical of the overall population.

## Conclusions

COVID-19-associated mucormycosis has a significant morbidity and fatality rate. The most essential factor in lowering mortality is to begin therapy as soon as feasible. Detection of raised CRP levels helps in prompt early initiation of anti-fungal treatment. Also, monitoring the levels of serum CRP will guide in deciding the time to stop the antifungals at an appropriate time. CRP monitoring is commonly available and affordable. CRP is an important biomarker in assessing the septic response to CAM. Hence, we recommend CRP monitoring of in-patients of mucormycosis on a global standard after further studies on a larger scale.
